# Supplementary material for: Loss of Meiotic Double Strand Breaks Triggers Recruitment of Recombination-independent Pro-crossover Factors in C. elegans Spermatogenesis
Source: bioRxiv. 2025 Jun 13:2025.06.10.658785. Preprint. [Version 1] doi: 10.1101/2025.06.10.658785 (PMC12259080; doi:10.1101/2025.06.10.658785)
Supplement: 1 [file NIHPP2025.06.10.658785V1-supplement-1.pdf]

bioRxiv preprint doi: https://doi.org/10.1101/2025.06.10.658785; this version posted June 13, 2025. The copyright holder for this preprint (which was not certified by peer review) is the author/funder, who has granted bioRxiv a license to display the preprint in perpetuity. It is made available under aCC-BY 4.0 International license.

Supplementary Figure Legends

Supplemental Figure 1. SPO-11-independent GFP::COSA-1 foci. A. Images of pachytene/condensation zones of male [*meIs8[unc-119(+) pie-1promoter::GFP::cosa-1]; spo-11(ok79)* germ lines imaged for GFP::COSA-1 fluorescence (green) and counterstained with DAPI (blue) in the absence (0 Gys) or presence (100 Gys) of IR. Bracketed region denotes faint GFP::COSA-1 foci in the [*meIs8[unc-119(+) pie-1promoter::GFP::cosa-1]; spo-11(ok79)* mutant in the absence of IR. B) Image of pachytene/condensation zones of *GFP::cosa-1(xoe44); spo-11(ok79)* males and L4 hermaphrodites undergoing spermatogenesis. White arrow denotes direction of meiotic progression. Bracket denotes region of germline with GFP::COSA-1 foci. Scale bar = 10 μm. C. Stacked bar graph showing percent nuclei with indicated numbers of GFP::COSA-1 foci in *GFP::cosa-1(xoe44)*; *spo-11(ok79)* males and L4 hermaphrodites. Three germlines were examined; 80 nuclei were scored in males and 58 nuclei were scored in L4 hermaphrodites. Number of GFP::COSA-1 foci: grey = 0; blue = 1; red = 2; purple = 3; neon green = 4; cyan = 5; rose = 6; gold = 7.

bioRxiv preprint doi: https://doi.org/10.1101/2025.06.10.658785; this version posted June 13, 2025. The copyright holder for this preprint (which was not certified by peer review) is the author/funder, who has granted bioRxiv a license to display the preprint in perpetuity. It is made available under aCC-BY 4.0 International license.

Supplemental Figure 2. The newly generated *xoe53* is a putative *rad-51* null allele. A. Top: Whole-mount hermaphrodite gonads of the indicated genetic backgrounds immunoassayed for RAD-51. Scale bar 20 μm. Color-coded insets (bottom left) show magnified early-mid pachytene regions to show no detectable RAD-51 foci in the *OLLAS::cosa-1; spo-11::AID::3xFLAG rad-51(xoe53) ieSi38* worms compared to *OLLAS::cosa-1; spo-11::AID::3xFLAG ieSi38* controls. Scale bar 5 μm. Representative images of DAPI-stained diakinesis nuclei depicting aberrant chromatin bodies in the *OLLAS::cosa-1; spo-11::AID::3xFLAG rad-51(xoe53) ieSi38* (-auxin) whose formation is suppressed upon SPO-11 depletion (+auxin). Scale bar 5 μm. B. Left: whole-mount male gonads of the indicated genetic backgrounds immunolabeled for RAD-51. Scale bar 20 μm. Right: magnified color-coded insets showing early-pachytene nuclei in control and *rad-51(xoe53)* mutant worms showing no detectable RAD-51 foci. C. Top: late-pachytene oocytes in the indicated genetic backgrounds and exposure conditions to auxin immunolabeled for OLLAS::COSA-1. Bottom: quantification of OLLAS::COSA-1 foci number in the same genotypes and exposure conditions to auxin. Scale bar 5 μm. Bars show mean with S.D. and asterisks denote statistical significance assessed by Kolmogorov-Smirnov test (*****p<0.0001, ns*= not significant). The number of nuclei analyzed in controls and *rad-51(xoe53)* are 122-172 (-auxin) and 130-160 (+auxin).

Supplemental Figure 3. RMH-1 is recruited at early and late meiotic stages in the male germ line. Whole-mount male germ line of the indicated genotype immunolabeled for GFP::RMH-1 and OLLAS::COSA-1, showing abundant RMH-1 foci formation in early pachytene and recruitment to presumptive CO sites together with COSA-1 in late pachytene cells. PMT: pre-meiotic tip; TZ: Transition Zone; EP: Early Pachytene; LP: Late Pachytene. Scale bar 20 μm.

bioRxiv preprint doi: https://doi.org/10.1101/2025.06.10.658785; this version posted June 13, 2025. The copyright holder for this preprint (which was not certified by peer review) is the author/funder, who has granted bioRxiv a license to display the preprint in perpetuity. It is made available under aCC-BY 4.0 International license.

Supplemental Figure 4. Recombination defects observed in *dsb-2* mutants are more severe in hermaphrodites than males. A. Whole-mount hermaphrodite and male germ lines dissected 48h post-L4 and immunostained for RAD-51/OLLAS::COSA-1 and counterstained by DAPI. Scale bar 20 μm. B. Color-coded insets showing magnified early and late pachytene cells from the indicated sexes depicting more robust recruitment of RAD-51 and COSA-1 in males versus hermaphrodites. Scale bar 5 μm.

Supplemental Figure 5. HR-independent COSA-1 recruitment in males does not depend on TOP-1 and TOP-2. A. Whole-mount male germ lines from the indicated genotype immunoassayed for TOP-1::AID::GFP and RAD-51. TOP-1 is efficiently depleted upon 24h exposure to auxin. Scale bar 20 μm. B. Quantification (top) and representative images (bottom) of OLLAS::COSA-1 foci in the indicated genotypes and exposure conditions to auxin in late pachytene nuclei. Bars show mean with S. D. and asterisks denote statistical significance assessed by Kolmogorov-Smirnov test (*****p<0.0001, ns*= not significant). The number of nuclei analyzed for *top-1::AID::GFP; OLLAS::cosa-1; spo-11::AID ieSi38* was 139 (-auxin) and 101 (+auxin). The number of nuclei analyzed for *top-2::AID; OLLAS::cosa-1; spo-11::AID ieSi38* was 138 (-auxin) and 100 (+auxin). Scale bar 1 μm.

bioRxiv preprint doi: https://doi.org/10.1101/2025.06.10.658785; this version posted June 13, 2025. The copyright holder for this preprint (which was not certified by peer review) is the author/funder, who has granted bioRxiv a license to display the preprint in perpetuity. It is made available under aCC-BY 4.0 International license.

Supplemental Table 1: Worm strains

Name	Genotype	Source
JEL993	*GFP(glo)::3xflag::cosa-1(xoe44) III*	Li *et al.*, 2022
JEL1369	*tra-2(e1095)/dpy-10(e128) unc-4((e120) II; GFP(glo)::3xflag::cosa-1(xoe44)III*	This study
JEL1367	*GFP(glo)::3xflag::cosa-1(xoe44) III; ced-3(ok2734) IV*	This study
JEL986	*[meIs8[unc-119(+) pie-1promoter::GFP::cosa-1] II; spo-11(ok79)/nT1 [unc-?(n754) let-?] (IV;V)*	Yokoo et al., 2012
JEL1395	*GFP(glo)::3xflag::cosa-1(xoe44) III; spo-11(ok79)/nT1 [unc-?(n754) let-?] (IV;V)*	This study
AV596	AV596 *cosa-1(tm3298)/qC1[qIs26] III*	Yokoo et al., 2012
CB4108	*fog-2(q71) V*	CGC
ERC84	*ers56[top-1::degron::GFP] I; ieSi57 [eft-3p::TIR1::mRuby::unc-54 3'UTR + Cbr-unc-119(+)] II*	Morao et al.; 2022
CA1199	*ieSi38 [sun-1p::TIR1::mRuby::sun-1 3´UTR + Cbr-unc-119(+)] IV*	CGC
NSV623	*OLLAS::cosa-1 III; spo-11::AID::3xFLAG rad-51(xoe53) ieSi38/nT1 (IV;V)*	This study
YKM240	*cdk-2::HA I*	Haversat et al.; 2022
NSV646	*cdk-2::HA I; OLLAS::cosa-1 III; spo-11::AID::3xFLAG ieSi38 IV*	This study
NSV420	*OLLAS::cosa-1 III; spo-11::AID::3xFLAG ieSi38 IV*	Hicks et al.; 2022
NSV688	*rmh-1 [syb9786 (GFP::rmh-1)] I*	This study
NSV662	*GFP::rmh-1 I; OLLAS::cosa-1 III; spo-11::AID ieSi38 IV*	This study
NSV97	*cosa-1 [ddr12(OLLAS::cosa-1)] III*	Janisiw et al.; 2018
NSV138	*OLLAS::cosa-1 III; him-5(ok1896) V*	Janisiw et al.; 2020
NSV690	*OLLAS::cosa-1 III; him-17(ok424)/nT1 (IV;V)*	This study
NSV694	*OLLAS::cosa-1 III; dsb-1(we11)/nT1 (IV;V)*	This study
NSV700	*dsb-2(me96); OLLAS::cosa-1*	This study
NSV676	*OLLAS::cosa-1 III; spo-11::AID::3xFLAG ieSi38/nT1; syp-2(ok307)/nT1 (IV;V)*	This study
JEL1393	*top-1::AID::GFP I; OLLAS::cosa-1 III; spo-11::AID::3xFLAG ieSi38 IV*	This study
ATG341	*top-2 [fq43(top-2::AID)] II; ieSi38 IV*	This study
NSV658	*top-2::AID II; OLLAS::cosa-1 III; spo-11::AID::3xFLAG ieSi38 IV*	This study

bioRxiv preprint doi: https://doi.org/10.1101/2025.06.10.658785; this version posted June 13, 2025. The copyright holder for this preprint (which was not certified by peer review) is the author/funder, who has granted bioRxiv a license to display the preprint in perpetuity. It is made available under aCC-BY 4.0 International license.

Supplemental Table 2: Antibodies used in this study

Antibody	Dilution	Source
Monoclonal Mouse anti-HA	1:600	BioLegend (#901501)
Polyclonal Rabbit anti-SYP-1	1:1000	Janisiw et al.; 2020
Polyclonal Rat anti-SYP-1	1:200	Hicks et al.; 2022
Polyclonal Rabbit anti-RAD-51	1:3000	Das et al.; 2022
Polyclonal Rat anti-RAD-51	1:500	Blazickova et al.; 2025
Polyclonal Rabbit anti-OLLAS	1:1000	Genscript (#A01658)
Monoclonal Mouse anti-GFP	1:500	Roche (#11814460001)
